# Can We Identify Patients in Danger of Complications in Retrograde Intrarenal Surgery?—A Retrospective Risk Factors Analysis

**DOI:** 10.3390/ijerph19031114

**Published:** 2022-01-20

**Authors:** Jakub Marek Ratajczak, Taras Hladun, Bartosz Krenz, Krzysztof Bromber, Maciej Salagierski, Michał Marczak

**Affiliations:** 1 Department of Management and Logistics in Health Care, Medical University of Lodz, 90-647 Lodz, Poland; michal.marczak@umed.lodz.pl; 2 Urology Department, Regional Specialized Hospital in Nowa Sól, 67-100 Nowa Sól, Poland; doc.hladun@gmail.com (T.H.); krenz.bartosz@gmail.com (B.K.); k.brom@wp.pl (K.B.); 3 Department of Urology, Collegium Medicum, University of Zielona Góra, 65-046 Zielona Góra, Poland; m.salagierski@cm.uz.zgora.pl

**Keywords:** RIRS, fURS, ureteroscopy, urolithiasis, kidney stone, complications, infection, UTI, sepsis

## Abstract

Retrograde intrarenal surgery (RIRS) is an innovative and effective method of kidney stones treatment, as it had great influence on the development of endoscopy in urology. The increasing prevalence of urolithiasis together with the rapid development of endourology leads to a rise in the number of procedures related to the disease. Flexible ureteroscopy is constantly being improved, especially regarding the effectiveness and safety of the procedure. The purpose of this study is to evaluate intraoperative and early post-operative complications of RIRS in the treatment of kidney stones. A retrospective analysis of medical records was performed. A series was comprised of 207 consecutive operations performed from 2017 to 2020. Complications occurred in 19.3% (*n* = 40) of patients. Occurrence according to the Clavien-Dindo scale was: 11.1% for grade I, 5.8% for grade II and 2.4% for grade IV. Infectious complications included SIRS (5.3%, *n* = 11) and sepsis (2.4%, *n* = 5). Statistical analysis revealed a correlation between acute post-operative infections and positive midstream urine culture, history of chronic or recurrent urinary tract infections, and increased body mass index (BMI). Furthermore, a significant correlation was observed between pain requiring the use of opioids with BMI over 25. Consequently, history of urinary tract infections, positive pre-operative urine culture, and increased BMI are considered risk factors and require appropriate management.

## 1. Introduction

Flexible ureteroscope (fURS) was used for the first time in 1983 by Bagley and collaborators, soon after the development of the modern semirigid ureteroscope in 1980 by Perez-Castro. However, major technological improvements have been made in the last two decades [1]. Although retrograde intrarenal surgery (RIRS) is a relatively new method of kidney stones treatment, it had a tremendous impact on the advancement of minimally invasive surgery in urology [2]. The paramount advantage of flexible scope over semirigid is the ability to inspect the whole kidney collecting system, enabling the opportunity to diagnose and treat not only stones, but also urothelial cancer [3]. The increasing prevalence of urolithiasis together with the rapid development of endourology leads to a rise in the number of procedures related to the disease [4]. European Association of Urology (EAU) recommends RIRS or shock wave lithotripsy (SWL) as first line treatment option for kidney stones of diameter up to 20 mm and second line treatment of stones over 20 mm [5]. Stones larger than 20 mm are qualified for percutaneous nephrolithotripsy (PCNL) mainly due to better irrigation fluid outflow, protecting from septic complications, and shortening operation time [6]. Small kidney stones of diameter less than 10 mm or medium size located in the lower pole of the kidney should primarily be treated with SWL.

Flexible ureteroscopy may be performed in various manners [7]. A typical technique was implemented by the authors: first, a safety guidewire is inserted into the ureter under fluoroscopy, followed by a hydrophilic access sheath, reaching to the upper ureter or uretero-pelvic junction, then the fURS is advanced to the kidney pelvis. Diagnostic ureteroscopy or prior double-J stenting may facilitate access to the ureter. Insertion of ureteral access sheath may cause ureteral trauma, however, pre-stenting lowers that risk [8]. Then ureteroscope is advanced to the kidney pelvis where lithotripsy can be performed, preferably using holmium:yttrium-aluminium-garnet (Ho:YAG) laser [9]. Stone disintegration types include dusting, which is used to produce powder of tiny fragments usually smaller than 2 mm, that are flushed spontaneously through the urinary tract; fragmentation, resulting in fragments that are removed with a basket [10]. After stone removal access sheath is withdrawn and a new double-J stent may be installed, if considered necessary.

Different types of lasers were implemented for endoscopic lithotripsy. Ho:YAG is the most commonly used device, in which energy ranges from 0.2 J to 6 J and 30 W power is considered a standard, however, merged generators can reach as high as 140 W [11]. Improvement made in recent years allowed for wide application of Ho:YAG laser, not only in urolithiasis, but also in prostate enucleation. Another advancement is the invention of the Moses effect, namely the division of laser pulse in two consecutive blasts creating a corridor of vapor in a fluid. The first forms a low attenuation cavity for the final energy impulse, therefore minimising power loss before hitting the target. As a result Ho:YAG amplified with Moses effect has a power of 120 W and frequency 5 to 80 Hz [12,13]. Recently introduced thulium fibre laser (TFL) is expected to revolutionise endourology by the ability to achieve a maximum power of 500 W, on average 60 W, alongside a very wide range of energy from 0.025 J to 6 J [14,15]. High frequency of impulses is an additional advantage of this technology, where peak values can reach 2400 Hz and regular dusting with an average frequency between 100 Hz to 200 Hz was enough to increase efficacy, without compromising complication rates [16,17]. The combination of these two features, high frequency, and low energy, facilitates major advances in dusting technique, which may produce colloid of particles smaller than Ho:YAG laser. Consequently, the time of lithotripsy can be reduced by half, in comparison to commonly used holium lasers. In addition, TFL has the smallest diameter, requiring minimal working channel, and enables the bending of endourological instruments [18,19,20]. A different laser incorporating this chemical element is Thulium: Yttrium-Aluminium-Garnet, however still being under investigation. This technology generates 120 W power, energy 0.1–3 J, and high frequency impulses up to 200 Hz, yet promises a low retropulsion effect due to long energy impulses [21,22].

EAU guidelines report an overall ureteroscopy complication rate of 9–25% [5]. Most of them are minor incidents, thus the procedure is generally considered to be safe. Nevertheless, major complications still occur, those of paramount importance are: acute urinary tract infection (UTI) and sepsis, ureteral avulsion or perforation, stricture of the ureter, vascular or enteric fistula formation, bleeding, cardiovascular events (i.e., stroke, pulmonary embolism), and death. Sepsis is defined as the dysregulated immune host response to infection triggered by simultaneous activation of proinflammatory cytokines, mainly tumour necrosis factor-α (TNF-α), interleukin (IL) 1 and 6, which are insufficiently suppressed by anti-inflammatory mediators including IL-4, IL-9, IL-10, epinephrine, transforming growth factor-β, soluble TNF-α receptors [23,24]. Sepsis mortality rates vary from 17.3% in general population and rise to 35.5%, if multi-organ dysfunction occurs [25]. Furthermore, intensive care generates a high cost of treatment causing a substantial financial burden for the healthcare system [26]. Emerging microbial resistance, especially the selection of new multi-drug resistant bacteria, decreases the effectiveness of antibiotic prophylaxis and empirical treatment. Eventually, it is estimated that 25% of patients with healthcare associated UTI develop sepsis [27]. Urosepsis is the most serious and possibly life-threatening complication of ureteroscopy [28,29].

Endoscopy of the upper urinary tract offers minimally invasive access via a natural orifice, however, requires caution and vigilance since ureteral tissues are very susceptible to injury [30,31]. Common technical mistakes are forceful manipulation of an instrument and accidental laser activation. These may lead to a mucosal lesion, perforation, or even avulsion of the ureter [8,32]. The trauma of the ureter frequently results in the formation of a false route or stricture, which warrants subsequent obstacles in the insertion of auxiliary equipment (guidewire, double-J catheter, access sheath). Perforated ureter, kidney pelvis, or ruptured calyx causes leakage of urine to the abdominal cavity, which may be a source of life-threatening infection. Management encompasses immediate repair or urinary drainage via nephrostomy or double-J catheter and delayed reconstruction if necessary. The main precautions in the prevention of upper tract injuries are the careful handling of equipment with subtle movements. Proper adjustment of instrument size is essential, further advancement of the ureteroscope, access sheath, guidewire, ureteral dilators cannot be forceful [33,34]. Stone extraction baskets and laser beams must only be employed under direct and unobscured vision [35].

The aim of this study is to evaluate the most significant peri-operative and early post-operative complications of retrograde intrarenal surgery in the management of urolithiasis. Another goal is to identify vulnerable patients at high risk of complications, who require special consideration and treatment strategy.

## 2. Materials and Methods

Retrospective analysis of consecutive medical records was performed. The data acquisition period spans from 2017 to 2020. Qualification criteria for RIRS were: single or multiple kidney stones, diameter ranging from 5 to 25 mm, and age over 18 years. Operations without lithotripsy were excluded, these represent mainly Randall’s plaques or calcifications not connected with kidney pelvis and ablation due to upper tract urothelial cancer. The procedure was carried out only on one side at a time. Many patients were referred after a series of unsuccessful SWL or because of multiple stones. The stone burden was evaluated by computed tomography and updated on ultrasound if shock wave treatment was applied. Double-J catheter was routinely installed, usually 2 weeks before the procedure. It was skipped in patients with a history of stent intolerance. In case of upsetting dysuria, bothersome haematuria, or very high intensity of physical work resulting in abdominal stent-related symptoms, the stent was removed. Mid-stream urine culture was taken prior to double-J insertion and routine antibiotic prophylaxis, or a full course of targeted therapy was administered according to reported bacterial susceptibility. Then the operation was planned on the last days of antibiotic treatment. Ciprofloxacin was first line prophylactic antibiotic until 2019, however, after European Medicines Agency (EMA) announcement was superseded by trimethoprim-sulfamethoxazole [36]. The vast majority of cases were completed by two urologists experienced in this surgical method, assisted by doctors in specialty training. Spinal anaesthesia was the preferred method of analgesia. Ureteral access sheath was used where possible. The following ureteroscopes were used in operations: fiberoptic Cobra Vision (Richard Wolf, Germany), FLEX-X2S (Karl Storz, Germany), and single-use digital Uscope PU3022, Pusen Medical Technology, Zhuhai, China). Lithotripsy was performed using a 30 W Ho:YAG laser with irrigation fluid being pumped manually. For safety reasons, operation time was limited to 60 min. Complications were classified according to Clavien–Dindo scale, which was adapted to evaluate endourological procedures [37]. Post-operative complications analysis comprised of occurrence of SIRS (systemic inflammatory response syndrome) and sepsis, nausea or vomiting, use of opioids, haematuria, mortality. The fever threshold was 38 °C. SIRS was defined by two or more of the following criteria: body temperature less than 36 °C or higher than 38 °C, heart rate greater than 90 beats per minute, a respiratory rate greater than 20 breaths per minute, and white cell count > 12,000/mm^3^ or <4000/mm^3^. Sepsis was defined as acute infection leading to organ dysfunction evaluated in biochemical parameters, elevated inflammatory markers, or Sequential Organ Failure Assessment (SOFA) score of minimum 2 points or at least 2 quickSOFA criteria: respiratory rate of 22 beats per minute or greater, altered mentation, or systolic blood pressure of 100 mm Hg or less [38]. Nausea/vomiting was identified by the use of antiemetic drugs. Follow-up encompasses events during hospitalisation. The success rate was based on endoscopy, ultrasound, and kidney-ureter-bladder X-ray, if useful. An evaluation was done on discharge day and defined as the absence of deposits of diameter up to 2 mm. 1. Post-operative pain was managed according to the World Health Organisation analgesic ladder. In the first step, all patients were administered paracetamol, 3 to 4 g daily. In the second step non-steroid anti-inflammatory drugs were given, usually ketoprofen or metamizole. At last tramadol, oxycodone or morphine was used to ease pain, the choice of medicine was at the discretion of the on-call physician. Sepsis was managed with empirical ceftriaxone or meropenem, fluids, and vasopressors in hemodynamically unstable patients. Intensive care was provided in the urology department with anaesthetists’ co-operation, since none of the patients required mechanical ventilation.

A univariate analysis was performed to compare the adverse events group with an uneventful group (Wilcoxon–Mann–Whitney-Test). A logistic regression analysis including patient’s age, body mass index (BMI), comorbidities, preoperative urine culture, stone size, and operation time as possible risk factors was completed using IBM SPSS Statistics Version 21 (International Business Machines Corporation, Armonk, New York, NY, USA). *p* < 0.05 was considered to indicate statistical significance.

## 3. Results

Overall, 207 records were included. The median age in the examined group was 58 years (SD = 13.7 years). 73% of patients were overweight, whereas the median BMI in the group was 27.8 (SD = 5.5). Study group characteristics are presented in Table 1.

**Figure 1:**
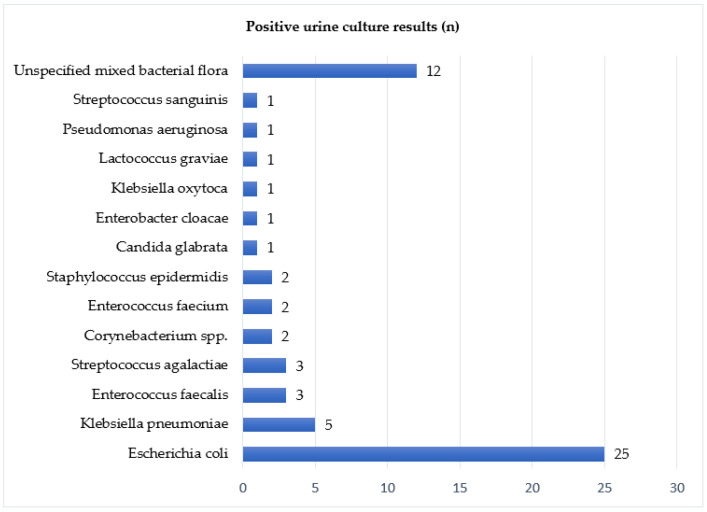
Positive urine culture results.

In order to facilitate ureteral access, a vast majority of patients (79%) were pre-stented with a double-J catheter before operation. Urine culture was taken routinely, and results were available for 88.4% of analysed records. Microbial growth was positive in 32.8% of samples. The most prevalent bacterium was *Escherichia coli* (41.7%), followed by miscellaneous bacterial flora (20%) and *Klebsiella pneumoniae* (8%). Table 2 contains urine culture results. Figure 1 presents a detailed incidence of bacterial strains.

Although EAU recommends flexible ureteroscopy for a maximum of 20 mm stones, indications were widened in two cases of slightly larger calculi i.e., 23 and 25 mm. These records were also included in order to follow the consecutive series. Stones from 5 to 15 mm dominate the group (88%), therefore the median size of stones was 10 mm. The median time of surgery was 45 min (SD = 15 min), and the 60 min threshold was intentionally minded. Usually, patients were admitted one day before the procedure and discharged the next day after surgery, if not presenting any signs of infection. As result, the average length of stay was two days (SD 1.7). 

Overall post-operative complications occurred in 19.3% (*n* = 40) of patients. Classification according to Clavien-Dindo scale is shown in Table 3. The most vital were infectious complications i.e., SIRS (5.8%, *n* = 12) and sepsis (2.4%, *n* = 5).

Statistical analysis revealed a significant correlation between that group and positive midstream urine culture (*p* < 0.01), as well as increased BMI and history of chronic or recurrent urinary tract infections (*p* < 0.01), ischemic heart disease (*p* < 0.01), and diabetes (*p* < 0.01). Neither operation time, nor stone size differentiated the occurrence of post-operative infection. Table 4 shows statistical significance of SIRS/sepsis risk factors. Although the size of the largest stone was irrelevant at 0.05 significance level, it was found significant at a level of 0.1 (*p* = 0.1). Incidence of nausea and vomiting (8.2%; *n* = 17) was statistically associated with BMI over 25 (*p* < 0.01), however was not influenced by operation time, nor the largest stone dimension. Pain requiring the use of opioids (i.e., tramadol, morphine, oxycodone) occurred in 4.8% of patients (*n* = 10). The research found a significant correlation of severe pain with increased BMI (*p* < 0.01), without association with length of operation or stone size. Clinically important intra- and post-operative hematuria occurred in one patient but did not require surgical intervention or transfusion. Neither reoperation nor deadly complications occurred. Complete lithotripsy was achieved in 80.6% of procedures.

Table 5 presents the estimated variance-covariance matrix and estimated correlation matrix is shown in Table 6. Most of the correlation between the variables is negligible or very small, which proves that the occurrence of given variables does not depend on the occurrence of another variable. Moderately significant relationships were found between the size of the largest deposit and the occurrence of UTI or diabetes, which indicates that deposits were greater in people with UTI or diabetes. BMI and UTI are correlated: in people with UTI, the BMI was on average higher. Finally, patients with diabetes are prone to UTI and urine culture was more likely to be positive in people with diabetes.

## 4. Discussion

Numerous studies aimed to assess common complications of ureteroscopy, both semirigid and flexible, to identify major or modifiable risk factors. Somani et al. in vast CORES URS Global Study conducted in 114 centres and involving 11,885 patients reported relatively low overall rate of complications (7.4%) and mortality (0.042%, *n* = 5), with only 3.0% of urinary tract infections (UTIs) [39]. Nevertheless, in that mixed group, the majority of ureteroscopies were semirigid, as most stones were located in the ureter, those in the kidney accounted for only 15.6% (*n* = 1852). Patients included in the CORES study who underwent retrograde intrarenal surgery for a single kidney stone were described by Skolariskos et al. Complication rates varied depending on the stone dimension, from 4.8% (*n* = 608) for stones less than 10 mm, 6.3% (*n* = 501) for stones 10–20 mm, reaching 9.3% (*n* = 54) for stones over 20 mm. The overall rate was 5.7%, in comparison to 19.3% in the presented analysis. Although this disparity could be striking, it may result from detailed reporting of minor events, such as pain and vomiting. This discrepancy lowers to 7.7% (5.8% and 2.4%, respectively) in the discussed study, whereas the incidence of serious infectious complications accounts for 3.6% (2.9% SIRS, 0.8% sepsis) in the research mentioned above.

Study sample comparable to this investigation, concentrating on 316 flexible ureteroscopies by Giusti et al. reported complications occurrence of 29.1%, meaningly higher than 19.3% found here. Nevertheless, the majority were minor events of Clavien-Dindo grade I in 17.4% (*n* = 55) and II in 9.5% (*n* = 30), with a very few grade III in 1.9% (*n* = 6) and grade IV in 1 patient (0.3%), without any of grade V [40]. Although the rates of class I (11.1%, *n* = 23) and II (5.8%, *n* = 12) in the presented research are lower, it may stem from the different clinical practices of medication usage, since these comprise of non-surgical management of complications. On the other hand, Berardinelli et al. in 377 RIRS observed 15.1% of adverse events, including 8.4% (*n* = 30) post-operative, which revealed even lower prevalence: grade I in 6.9% (*n* = 26), grade II in 0.5% (*n* = 2), grade III in 0.5% (*n* = 2) [41]. Similarly, a Swedish study of 486 mixed semirigid and flexible URS groups by Wagenius et al. described: grade I in 12.0% (*n* = 68), grade II in 6.5% (*n* = 7), grade III in 1.9% (*n* = 11) and grade IV in 0.2% (*n* = 1), which are comparable to the incidence of adverse events in the sample being discussed [42].

A recent systematic review by Chugh et al. states post-URS complications, both semirigid and flexible, in 7.9% (*n* = 1919), of which 3.9% (*n* = 972) were infectious, including sepsis in 0.51%, and 4% (*n* = 1147) were non-infectious. According to the Clavien-Dindo scale these were grade I in 5.3% (*n* = 1298), grade II in 1.1% (*n* = 275), grade III in 0.7% (*n* = 180), grade IV in 0.3% (*n* = 91), and grade V complications only in 0.0001% (*n* = 3) patients [43]. Major risk factors of UTI are positive preoperative urine culture, diabetes, female gender, indwelling ureteric stents, stone burden, and operation time [44,45,46]. Although women are more susceptible to urinary tract infections and subsequent recurrences, in the presented study female gender was found to be irrelevant regarding the occurrence of SIRS or sepsis [47,48].

Another research of 3298 standard URS by Southern et al. resulted in 6.9% of post-operative fever or SIRS, similar to a study by Baboudijan et al. where UTI occurred in 6.7% [49,50]. A large cohort analysed by Senocak et al. revealed that 22.1% of 492 patients had post-operative complications after fURS, in total infectious complications occurred in 8.5% [51]. The aforementioned rate is slightly higher than the group investigated herein (7.7%), however, this occurrence is considered comparable. In Berardinelli et al.’s research, infectious complications were recorded in 7.7% of patients, mainly consisting of fever in 4.4%, SIRS 1.7%, and sepsis in 0.7% [52]. In another paper, Blackmur et al. reported 7.4% urosepsis within 28 days after the operation and positive preoperative urine culture was significantly associated with post-operative urosepsis, despite the use of preoperative antibiotic treatment [53]. In the presented research midstream urine culture, together with a history of recurrent or chronic UTI, was confirmed as a risk factor for septic complications. Preoperative urine samples were positive in 68.7% of patients diagnosed with SIRS and sepsis, indicating asymptomatic bacteriuria. Targeted therapy was administered before the operation, without curative intention, rather that reduction of urinary microbiota. Regarding the presence of stones covered with biofilm, rapid recurrence after cessation of the antimicrobial agent was considered highly probable. Antibiotic treatment was continued after the operation and extended in the suspicion of sepsis, with the use of additional meropenem or third generation cephalosporins. Vigilance and rapid response to the development of systemic symptoms, confirmed by the rise of inflammation biomarkers (C-reactive protein or procalcitonin), resulted in a complete reduction of the mortality rate. Studies investigating the incidence of post-operative UTI in patients with a history of sepsis or pyelonephritis brought inconsistent results. On one hand, Youssef et al. found that elective ureteroscopy after successful management of sepsis was associated with a higher overall complication rate, prolonged hospital stay, and longer antibiotic administration [54]. On the other hand, in later studies, the previous acute infection did not escalate the risk of adverse events [55,56].

In the group being presented double-J catheter was inserted usually within two to four weeks before RIRS. Preoperative ureteral stenting decreased the rate of septic events in the group reported by Blackmur. Nevertheless, it was not confirmed in this group, since all patients diagnosed with sepsis were routinely pre-stented with adherence to standard protocol applied in the department. Sepsis rates are correlated with the duration of stent dwelling. Nevo et al. revealed that the presence of ureteral stent for one, two, three, and over three months elevated the risk of sepsis to 1, 4.9, 5.5, and 9.2%, respectively [57]. Clearly, it has been proven that the ureteric catheter should be kept as short as possible before ureteroscopy, preferably up to two weeks.

Ureteral access sheath was employed routinely to facilitate the outflow of irrigation fluid and minimise intrarenal pressure [58]. Reported pressure values range from 40 cm H_2_O to as high as 199 cm H_2_O during pumping. Implementation of ureteral access sheath could lower it to less than 30 H_2_O mm passive irrigation, yet may still reach 200 mm H_2_O when flushing [59]. Although the role of access sheath remains uncertain, the article by Traxer et al. supported its use to reduce the rate of infectious complications. Another cohort described by Geraghty et al. did not confirm that hypothesis in the treatment of renal stones over 20 mm diameter, however, indicated that high stone burden also increases the risk of sepsis [60]. Higher occurrence of post-operative fever or UTI in stones larger than 20 mm was confirmed by Skolarikos et al. since dimensions affect operation time and may prolong exposure to elevated intrarenal pressure [61].

Taking this into account at the stage of qualification for the procedure, a general prerequisite for the maximum size of 20 mm was made, with only a few exceptions, not exceeding 25 mm in the largest dimension. Consequently, in the investigated group stone size is not correlated with SIRS nor sepsis, which retrospectively validates sound qualification criteria. This finding reinforces recommended thresholds of acceptable stone burden. On top of that, dynamic technological progress in the construction of more powerful lasers and thinner scopes may mitigate known risk indicators and again move acknowledged limits much further.

The age of patients was found to have no impact on adverse events. Research in elderly patients has proven the safety of RIRS, with similar complication rates and results, as in younger patients [62]. Infectious adverse events in the analysed cohort were correlated with ischemic heart disease and diabetes, however, these comorbidities were mutually correlated, indicating that diabetes contributes to heart ischemia. Although the incidence of urinary tract infections is higher in diabetic patients, in other studies it was not a statistically relevant risk factor of SIRS or sepsis after flexible ureteroscopy [63]. Lately published research revealed that previous history of fluoroquinolones may also pose a threat of drug-resistant bacterial infection [64]. In addition, the albumin to globulin ratio can serve as an infection predictive factor, as it may indirectly reflect immune competence [65].

Significant post-operative haematuria resulting in prolonged hospital stay occurred in only 1 patient. The bleeding ceased after pharmacological treatment, without surgical intervention or blood transfusion. The possible sources of haemorrhage are mucosal injury of kidney pelvis, caused by stone fragments, or friction of access sheath against the wall of the ureter, still, these resolve spontaneously. Among available modalities of urinary stone treatment, flexible ureteroscopy in the only method acceptable in compromised coagulation and was proved safe with a reported bleeding rate of 4%, generally described as minor [66,67]. In comparison to perioperative blood loss and transfusions, RIRS presents a clear advantage over PCNL [68,69].

Opioids (i.e., tramadol, morphine, oxycodone) were administered when non-steroidal anti-inflammatory drugs (NSAIDs) did not sufficiently relieve symptoms. Post-operative pain requiring the use of opioids was recorded in 4.8% of patients, Oguz et al. found substantial pain in 18.4% after RIRS, whereas in the CROES study clinically noteworthy pain occurred very rarely (0.33%) [39]. The presence of symptoms was significantly correlated with increased BMI (*p* < 0.01), however, was not associated with operation time or stone burden, contrary to findings of Oguz et al. who pointed female gender, higher stone burden, and length of access sheath as contributing factors. Antiemetic medicines were given in 8.2%, usage was more frequent in overweight and obese patients, statistically associated with BMI over 25 (*p* < 0.01). In the CROES study, nausea and vomiting were less frequent (0.03%). Relatively higher incidence of mild complications in the discussed analysis might result from physicians’ susceptibility to pharmacological interventions. Evidence addressing the impact of obesity on post-operative pain and emesis in ureteroscopy is scarce, thus should require further investigation.

In the presented study increased BMI (over 25 kg/m^2^) was also found to be a risk factor of complications in general. Based on the CROES cohort Krambeck et al. reported a higher incidence of post-operative adverse events in underweight and extremely obese patients, along with the need for additional procedures [70]. Assumption can be made there may have been undiagnosed diabetes or improper fasting glucose in some patients with higher BMI, leading to immune suppression. There are also not yet clearly identified mechanisms of chronic inflammation in obese patients additionally jeopardising immunity. Despite relatively elevated perioperative risk and lower efficacy, ureteroscopy is considered safe in obese patients [71,72].

The occurrence of complications regarding the experience of urologists was evaluated by Komori et al. Study found that climbing the learning curve required a longer operation time and resulted in a lower stone-free rate, together with an increased risk of ureteral injury. Sufficient knowledge of the operation was estimated to be achieved after around one hundred procedures at a high-volume centre. It is noteworthy to state that the incidence of sepsis was unrelated to the surgeon’s experience and occurred at a similar rate [73].

All RIRS procedures analysed in this paper were planned, elective operations. Still, the need for expanding access to flexible ureteroscopy must be underlined, especially in order to avoid unnecessary readmissions, multiple shockwave therapy sessions, and further semirigid operations [74]. Constant improvement of single-use devices leads to gradual price decline and enhanced cost-effectiveness, especially in low-volume centres. Reusable scopes remain the best option for experienced operators, assuming a low damage rate and high durability of the instrument [75,76].

Having considered the limitations of the study, it should be noted that research was conducted retrospectively with a focus on perioperative complications, which occurred during the hospital stay. Furthermore, as long-term follow-up data was limited, lithotripsy effectiveness was based on endoscopic, ultrasound, and X-ray evaluation. The department serves as a stone centre for a wide area, thus the majority of patients received further control in remote practices, and data on long term follow-up was not substantial enough to draw statistical conclusions. Urine culture results were not included in 11.6% of analysed medical records. Application of Clavien–Dindo scale may not comprehend some self-limiting complications.

## 5. Conclusions

Treatment of kidney stones using flexible ureteroscopy is generally safe and effective, however, rare life-threatening complications may still occur. Significant risk factors for septic complications include a positive midstream urine culture, history of chronic or recurrent urinary tract infections, increased BMI, and diabetes. The research found a significant correlation of opioid pain treatment with an increased BMI. Careful qualification and consideration of risk factors in preparation for surgery minimises potential perioperative risks, therefore, midstream urine culture should be considered obligatory before flexible ureteroscopy.

## Figures and Tables

**Table 1:** Study group characteristics.

Parameters	% or SD Value	
Age (years)	58 (13.7)	
Sex	Women 53.4%	
	Men 46.6%	
BMI (kg/m^2^)	27.8 (5.5)	
Operation side	Left 50.7%	
	Right 49.3%	
Operation time (min)	45 (15)	
Stone size (mm)	Occurrence	Size (SD)
Largest stone	100%	10 (3.9)
2nd	37.20%	6 (2.8)
3rd	12.60%	5 (1.9)
Largest stone location:		
Upper calyx	11.80%	
Medium calyx	21.10%	
Lower calyx	45.60%	
Kidney pelvis	21.60%	
2nd Largest stone location:		
Upper calyx	10.40%	
Medium calyx	40.30%	
Lower calyx	37.70%	
Kidney pelvis	11.70%	
Comorbidities:		
Ischemic heart disease	8.6% (*n* = 18)	
Diabetes	15.9% (*n* = 33)	
Recurrent/chronic UTI	20.3% (*n* = 42)	
Chronic kidney disease	4.3% (*n* = 9)	
Hypertension	23.2% (*n* = 48)	
Hypothyroidism	8.2% (*n* = 17)	
Gout	4.8% (*n* = 10)	
Chronic obstructive pulmonary disease	2.4% (*n* = 5)	

**Table 2:** Mid-stream urine culture results.

Mid-Stream Urine Culture:	% (*n*)
Total Available	88.4% (183)
Negative/sterile	67.2% (123)
Positive	32.8% (60)
Not available	11.6% (24)

**Table 3:** Results and description of surgical complications according to Clavien-Dindo classification.

Grade	Description	*n*	%
I	Any deviation from the normal post-operative course without the need for pharmacological treatment or surgical, endoscopic and radiological interventions. Allowed therapeutic regimens are: drugs as antiemetics, antipyretics, analgesics, diuretics and electrolytes and physiotherapy. This grade also includes wound infections opened at the bedside.	23	11.1
II	Requiring pharmacological treatment with drugs other than such allowed for grade I complications. Blood transfusions and total parenteral nutrition are also included.	12	5.8
III	Requiring surgical, endoscopic or radiological intervention.	null	null
IV	Life-threatening complications (including CNS complications) requiring IC/ICU-management.	5	2.4
V	Death of a patient.	null	null

**Table 4:** Parameters affecting occurrence of SIRS/sepsis.

	Level of Effect	Odds Ratio	Lower CI 95.0%	Upper CI 95.0%	*p*
Largest stone diameter (mm)		0.878796	0.760866	1.01500	0.078850
Normal weight	BMI 18.5–25	2.749022	0.579156	13.04850	0.142517
Underwieght	BMI < 18.5	0.216355	0.013141	3.56211	0.119581
Overweight	BMI > 25	2.526093	0.630934	10.11381	0.148003
UTI		4.852125	1.308780	17.98860	0.018153
Diabetes		2.977971	0.635067	13.96436	0.166329
Urine culture	Non-sterile	0.139426	0.041352	0.47010	0.001487

**Table 5:** Estimated variance-covariance matrix.

	Intercept	Largest Stone Diameter (mm)	BMI 18.5–25	BMI < 18.5	BMI > 25	History of Infection	Diabetes	Positive Urine Culture
Intercept	0.980160	−0.062800	−0.001948	0.316145	−0.118121	0.045744	−0.024435	−0.008929
Largest stone diameter (mm)	−0.062800	0.005405	−0.004559	−0.000998	0.001139	−0.008407	−0.006558	0.000237
BMI 18.5–25	−0.001948	−0.004559	0.384616	−0.394674	−0.003049	0.021113	−0.015871	−0.022897
BMI < 18.5	0.316145	−0.000998	−0.394674	1.100130	−0.370720	−0.043936	−0.018200	0.030884
BMI > 25	−0.118121	0.001139	−0.003049	−0.370720	0.325158	0.012591	0.016305	−0.021175
History of infection	0.045744	−0.008407	0.021113	−0.043936	0.012591	0.111738	0.043180	−0.010176
Diabetes	−0.024435	−0.006558	−0.015871	−0.018200	0.016305	0.043180	0.155400	−0.017252
Positive urine culture	−0.008929	0.000237	−0.022897	0.030884	−0.021175	−0.010176	−0.017252	0.096138

**Table 6:** Estimated correlation matrix.

	Intercept	Largest Stone Diameter (mm)	BMI 18.5–25	BMI < 18.5	BMI > 25	History of Infection	Diabetes	Positive Urine Culture
Intercept	1.000000	−0.862795	−0.003173	0.304449	−0.209234	0.138224	−0.062608	−0.029089
Largest stone diameter (mm)	−0.862795	1.000000	−0.099989	−0.012945	0.027170	−0.342084	−0.226265	0.010375
BMI 18.5–25	−0.003173	−0.099989	1.000000	−0.606740	−0.008621	0.101843	−0.064918	−0.119077
BMI < 18.5	0.304449	−0.012945	−0.606740	1.000000	−0.619836	−0.125313	−0.044017	0.094964
BMI > 25	−0.209234	0.027170	−0.008621	−0.619836	1.000000	0.066057	0.072534	−0.119763
History of infection	0.138224	−0.342084	0.101843	−0.125313	0.066057	1.000000	0.327683	−0.098180
Diabetes	−0.062608	−0.226265	−0.064918	−0.044017	0.072534	0.327683	1.000000	−0.141146
Positive urine culture	−0.029089	0.010375	−0.119077	0.094964	−0.119763	−0.098180	−0.141146	1.000000

## Data Availability

The data presented in this study are available on request from the corresponding author. The data are not publicly available due to healthcare facility policy.
